# Mosquitoes Associated with Ditch-Plugged and Control Tidal Salt Marshes on the Delmarva Peninsula

**DOI:** 10.3390/ijerph8083099

**Published:** 2011-07-25

**Authors:** Paul T. Leisnham, Sarah Sandoval-Mohapatra

**Affiliations:** Department of Environmental Science and Technology, University of Maryland, College Park, MD 20742, USA; E-Mail: sarah.sandoval.mohapatra@gmail.com

**Keywords:** ditch plugging, Delmarva Peninsula, tidal salt marsh, restoration, salinity

## Abstract

A study was conducted during the summer of 2009 (from July to September) to characterize mosquito communities among different habitats in five historically ditched tidal salt marshes and three adjacent wooded areas in the E.A. Vaughn Wetland Management Area on the Maryland Delmarva Peninsula, USA. Study marshes are characteristic of Atlantic coastal salt marshes that had undergone grid ditching from the 1930s to 1950s. In the autumn of 2008 (October and November) ditches were plugged near their outlets in two (‘experimental’) marshes with the aim to restore their natural tidal hydrology. The three other marshes were not plugged. Marshes were sampled from July to September in 2009 by using standard dip count method. A total of 2,457 mosquito larvae representing six species were collected on 15.4% (86/557) of all sample occasions and 399 adults representing four mosquito species were collected from landing counts. *Aedes sollicitans*, *Anopheles bradleyi* and *Culex salinarius* were the most common species collected in larval habitats, and *Ae. sollicitans* was the most common adult collected. Wooded habitats had more total mosquitoes, were also more frequently occupied by mosquitoes and had higher densities of mosquitoes than marsh habitats. Almost all larvae collected from marshes were from one experimental and one control site. The majority of larvae at the control site were *Ae. sollicitans* in marsh pannes while *Cx. salinarius*, *An. bradleyi*, *Ae. cantator*, *and Ae. sollicitans* were collected in high numbers from ditches at the experimental site. We found a difference in the proportion of marsh pannes occupied by *Ae. sollicitans* but not total mosquitoes sampled 4–5 days after spring tide events than on other occasions. Salinity measures of 42 larval habitats showed lower median salinity in mosquito-occupied habitats (11.5 ppt) than unoccupied habitats (20.1 ppt), and in habitats in wooded areas followed by ditches and pannes in marsh areas. The results of this study suggest that wooded areas adjacent to salt marshes may be a substantial source of biting adult mosquitoes usually associated with salt marsh habitats and that ditch plugging may alter the productivity of mosquitoes on some marshes. We recommend future studies consider mosquito productivity from habitats surrounding salt marshes, and if assessments of marsh alterations are a goal, compare multiple experimental and control areas before and after treatments to determine if alterations have a consistent impact on regional mosquito production.

## 1. Introduction

Coastal salt marshes are among the most common and productive coastal habitats on Earth and they play an important ecological role in the interface between marine and terrestrial environments [1]. Salt marshes are also important for public health worldwide as larval habitats for disease-vector mosquitoes and significant biting pests (e.g., [2–5]). Under the direction of economic-rebuilding “New Deal” initiatives, the Civilian Conservation Corps dug an estimated 562,000 miles of parallel ditches in ≈90% of the coastal salt marsh from Maine to Virginia [1,6]. Ditches were intended to control mosquito production by draining shallow pooled waters called pannes where mosquito larvae commonly develop and by allowing fish to feed on mosquito prey during high tides. However, there is considerable evidence that grid ditching may negatively impact the ecology of salt marshes by changing the vegetation composition [6], and decreasing habitat quality for semiaquatic invertebrates [1,6], fish [7], and wildlife [8]. With the aim of helping restore the natural ecology of salt marshes and to use more benign mosquito management practices, some conservation efforts have started to plug ditches [9]. Ditch plugging is expected to help marshes hold water in a more natural way during normal tidal flow and thus restore natural ecological functions [9]. Marsh conservation and mosquito control has usually focused on the effectiveness at controlling mosquito production of open marsh water management (OMWM), which is characterized by the targeted construction of tidal channels, ponds, and shallow radial ditches [10]. Despite recent ditch-plugging projects, only one study in New England has characterized mosquito communities in marshes that have plugged ditches with comparable marshes without plugging (or ‘control’ marshes) [4]. This study sampled larvae among treatment and control sites but only identified a small subsample of collected individuals and reported no conclusions on the relative densities of mosquito species among marsh areas or neighboring habitats [4].

Mosquito species that breed in salt marshes along the Atlantic coast of the USA can be a considerable nuisance to humans and are likely to vary in their importance in transmitting a range of arboviruses, including eastern equine encephalitis virus (family *Togaviridae*, genus *Alphavirus* EEEv) and West Nile virus (family *Flaviviridae*, genus *Flavivirus* WNv). *Aedes sollicitans* is considered the most common saltmarsh mosquito species in the region and is the main vector of epidemic EEEv [11,12], but there is usually considerable local and regional variation in mosquito communities that may have important implications for human disease risk. For example, *Culex salinarius* is often collected from saltmarsh habitats and can vector EEEv [13], but is also considered important in WNV transmission to humans [14]. *Anopheles bradleyi*, which is a common coastal species of the *An. crucians* complex, is also an important vector of EEEv and WNv [15].

EEEv was first detected in the USA in the 1930s and although relatively rare (only 260 symptomatic cases from 1964–2009 [16]), it causes 50–75% mortality from encephalitic infections, making it the most severe insect-borne disease in North America. In the last five years, EEEv virus transmission has intensified throughout the northeast US and Canada, and spread to areas where it had not been previously detected [17]. Since its invasion in 1999, WNV has infected over 30,000 people and caused over 1,200 deaths in the USA to represent the most medically important mosquito-borne disease in North America [18]. Given the emergence of EEEv and WNv, it is increasingly important to understand how habitat manipulations may affect vector communities in coastal regions.

Disease-vector mosquitoes that breed in salt marshes may differentially colonize less saline ground pools in adjacent upland sites making it difficult to determine the larval source of biting and vector adults in an area [19,20]. For a better understanding of potential larval production in salt marshes it is necessary to characterize mosquito communities across different habitats in plugged and control marshes, and adjacent upland sites in focal areas. A preliminary study in 2009 was conducted to compare mosquito communities in different habitats among marsh sites that were ditch-plugged by the Maryland Department of Natural Resources in the autumn of 2008 (October and November) with neighboring control marshes and adjacent upland sites. This study was part of a broader restoration program focused on returning the ecology of salt marshes back to a more original state, and would provide valuable data to help future monitoring in the area.

## 2. Experimental Section

### 2.1. Study Sites

The marshes selected for this study are associated with the E.A. Vaughn Wetland Management Area (WMA), on MD Route 12 in southeastern Worcester County, MD, USA. Worchester County is located on the middle portion of the eastern side of Delmarva Peninsula and is mainly rural, with a variety of freshwater and saline systems. Vegetation at all sites represents a typical coastal plain salt marsh [21] with a predominately *Spartina alterniflora* zone merging into an upland *S. patens* zone (Maryland Department of Natural Resources, unpublished data). Mixed needled and broadleafed evergreen woodlands bordered the upland sides of all marsh sites.

**Figure 1.**
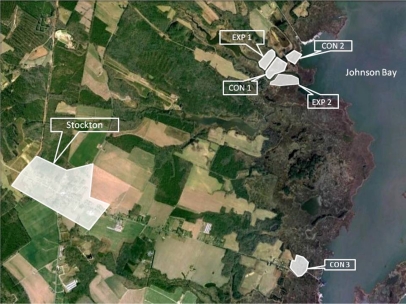
Aerial photograph of study sites on the Maryland Delmarva Peninsula. EXP1, EXP2, CON2 and CON3 are separated by natural features and independent hydrologic units, while EXP1 and CON1 are separated by a ditch and not hydrologically independent.

In the autumn of 2008, the Maryland Department of Natural Resources inserted 2.0–3.0 meter peat plugs sourced from the marsh with polythene backings near the outlets of all ditches in two salt marshes (EXP1 and EXP2). To compare the effects of ditch plugging with reference conditions, three neighboring ‘control’ salt marsh areas (CON1, CON2, and CON3) were also selected for this study. EXP1, EXP2, CON1, and CON2 are located in Johnson Bay (38.07°N; −75.37°W) (Figure 1).

CON3 is located approximately 3 km south along MD Route 366 (38.04° N; −75.37°W; Figure 1). EXP1, EXP2, CON2, and CON3 are separated from each other by natural features (*i.e.*, an upland peninsula or natural tidal creek) and thus enable comparisons between independent hydrologic units. CON1 is immediately downstream of EXP1, thus CON1 and EXP1 are not independent hydrological units. Comparisons between EXP1 and CON1 allow an examination of the effect of ditching at one site on a neighboring marsh area. The close proximity of study sites means that they experience similar hydrologic regimes and minimal intrinsic marsh differences. All sites are also within the typical flying range of saltmarsh mosquitoes and have been observed to have high mosquito production during past seasons (D. Webster, Maryland Department of Natural Resources, *personal communication*).

### 2.2. Larval Mosquito Sampling

Larval collections were conducted approximately weekly (nine sampling trips) from July to September 2009, which is the period of greatest mosquito activity. The aim of our sampling was to collect and identify all collected larvae among different habitats types to characterize mosquito communities in salt marshes rather than sample over a longer period with less intensity over fewer habitat types as in past studies (e.g., [20]). Four sampling trips (28 July, 11 August, 25 August, 11 September) were 4–5 days after spring tide events when pannes are likely to be flooded. Sampled areas at each site extended from the upland woods approximately 150 m towards the tidal source and ditch plugs at EXP 1 and EXP 2. Approximate sizes of each site were as follows: EXP1, 46.2 ha; EXP2, 41.0 ha; CON1, 16.5 ha; CON2, 15.9 ha; CON3, 42.7 ha. Larval mosquitoes were sampled using a 400-mL dipper and mosquito production quantified using the standard dip count method with densities expressed in numbers per dip. During each collection trip, pannes, ditches, and permanent ponds in marsh sites, and ground pools within wooded areas were sampled. Transects that traversed the elevation gradient from the treeline to the tidal source were randomly established through marsh sites each trip. At approximately 30-m intervals along all transects, the nearest shallow panne within a 3-m radius was located and sampled by taking 5–6 dips. Ditches were randomly selected and each ditch was sampled within a 10-m long stretch. Because EXP1, EXP2 and CON3 were larger areas compared to CON1 and CON2, we sampled approximately twice as many collection sites at these sites from July to September (summer) (Table 1). Ground pools in three wooded areas that consisted on bordered the upland edges of the marsh sites were also sampled. One area (EXP1-CON1-CON2) was part of a contiguous upland peninsula bordering EXP1, CON1, and CON2, while the other two wooded areas bordered EXP2 and CON3 marsh sites respectively. All wooded areas were approximately 15 ha in size and were sampled by randomly selecting ground pools while walking through them. Numbers of dips in each habitat in marsh and wooded areas were proportional to the size of the habitat so that sampling effort was approximately even among individual habitats. This sampling design resulted in non-biased sampling of mosquito communities in all marsh sites and neighboring wooded habitats [22]. Between 45–90 pannes, 9–24 ditches, and ponds were sampled over the duration of summer sampling. Ponds were also sampled at most sites except CON2, which did not have any ponds. EXP1, CON1, and CON3 had only 1 large pond thus samples were taken from the same pond and cannot be treated as independent. Because ditching is likely to affect water salinity and because salinity affects the abundance and composition of mosquitoes, a subset of collection habitats with a range of mosquito densities were measured for their salinity over 5 sampling trips using an YSI hand held meter. Rainfall over the five days preceding each sampling trip was collected from the closest meteorological station (Snow Hill, <4.4 km from study sites) of the U.S. National Climate Data Center (http://www.ncdc.noaa.gov/oa/mpp/freedata.html). Samples from each collection site were stored individually in 70% ethanol for later identification using standard keys [23]. Early-instar specimens were first raised to 3rd or 4th instar before storage to aide identification.

### 2.3. Adult Mosquito Sampling

Adult landing count collections were obtained on the marsh sites and in the wooded areas three days, from September 11 to 13 2009. Adult counts were taken during dusk (5:00–6:30 p.m.) by collecting them immediately on exposed arms of a researcher (PL) for periods of 4–10 minutes. Collections were made using a C-cell flashlight aspirator equipped with 13mm of flexible vinyl tubing and a 59.1-cc polystyrene vial. Adults were dry and stored in the lab for later identification using standard keys [23]. Species in the *An. crucians* complex are indistinguishable as adults but because all *An. crucians* complex larvae that we collected were *An. bradleyi*, we assumed that all adults were *An. bradleyi* and recorded that as this species.

### 2.4. Data Analysis

Frequencies of occupied habitats were compared between sites (pooling habitat types and dates), habitat types (pooling sites and dates) and sample trips (pooling habitat types) by Chi-squared tests [24]. Relationships between rainfall with total mosquitoes and *Ae. sollicitans* in marsh pannes among sample trips was tested using regression analyses (pooling sites) [24]. Median differences in salinity were compared between mosquito-occupied and unoccupied habitats using Kruskall-Wallis Test [24]. All analyses were done using SAS [25] using experiment-wise α = 0.05.

## 3. Results and Discussion

### 3.1. Larval Mosquito Sampling

A total of 2,457 mosquito larvae representing six mosquito species were collected during 15.4% (86/557) of all sample occasions. The total number of mosquitoes collected in wooded habitats (61.7%, 1,515/2,457) were more than in saltmarsh habitats (38.3%, 942/2,457). *Aedes sollicitans* was the most frequent species collected overall (45.7%, 1,124/2,457) and in wooded habitats only (57%, 864/1,515), while *Anopheles bradleyi* was the most frequent species collected in marsh habitats only (34.1%, 321/942) (Tables 1 and 2). Overall, *Ae. taeniorhynchus* was the second most frequent species collected (13.1%, 321/2,457) but was restricted almost entirely to wooded areas (Tables 1 and 2). Mosquitoes were consistently collected more frequently and in greater densities from wooded ground pools compared to marsh habitats (Tables 1 and 2). Because ponds were not represented among all sites and few mosquitoes were collected from them (1.3%, n = 31) we only included data from panne, ditch, and wooded habitats in further analyses.

Marsh sites had different frequencies of mosquitoes (pooling panne and ditch data: χ^2^_4_ = 92.7, P < 0.001), with EXP1 and CON1 having the highest proportions of sample locations occupied by mosquitoes among all sites (Table 1). Almost all larvae collected from marsh habitats were from EXP1 and CON1 (99.6%, 907/911), but the mosquito species and type of occupied habitats differed between these two sites. The majority of larvae (80.3%, 208/259) from CON1 were collected from pannes while the majority (79.0%, 512/648) of larvae from EXP1 was collected from ditches. *Aedes sollicitans* was the most frequent species collected in pannes at CON1 (76.4%, 159/208) and across all sites (54.5%, 190/348). *Culex salinarius* (44.7%, 229/512) was the most frequent species collected in ditches in EXP1 but was not collected in ditches at other sites. EXP1 had the highest species diversity among marsh sites with *An. bradleyi* (25.0%, 128/512), *Ae. canator* (17.4%, 89/512), and *Ae. sollicitans* (12.9, 66/512) all being collected in relatively high numbers in ditches (Table 1).

There were no differences in the proportions of sample locations occupied by mosquitoes between wooded areas (χ^2^_2_ = 4.4, P < 0.109) but fewer species and total mosquitoes were collected from the wooded area bordering CON3 compared to the other two areas (Table 2). All six species collected in this study were represented in the wooded areas bordering EXP2 (Table 2), including *Psorophora ferox* which was never collected in other wooded areas or marsh habitats (Table 1). The proportion of mosquito-occupied habitats varied among sampling trips across all combined habitat types (χ^2^_8_ = 21.7, P < 0.005) and wooded habitats only (χ^2^_8_ = 37.8, P < 0.001), but not marsh habitats only (χ^2^_8_ = 12.7, P < 0.117). We found a difference in the proportion of pannes occupied by *Ae. sollicitans* (χ^2^_1_ = 8.74, P < 0.003), but not total mosquitoes (χ^2^_1_ = 0.22, P < 0.637), between habitats sampled within 5 days after spring tide events and the habitats sampled on the remaining sampling trips, with more habitats occupied by *Ae. sollicitans* after tides. We found no evidence that the proportion of pannes or wooded pools occupied by total mosquitoes or *Ae. sollicitans* changed with rainfall over the five days preceding sampling trips (regression: F_1,7_ = 0.01–1.93, P-values = 0.207–0.925).

Salinity was measured in 13 habitats with mosquitoes and 29 habitats without mosquitoes across all sites. Median salinity was lower in mosquito-occupied habitats (11.5 ppt) compared to unoccupied habitats (20.1 ppt) (H_1_ = 8.01, P = 0.005). Salinity also varied between habitat types (H_2_ = 10.21, P = 0.006), with the highest median salinity in pannes (19.2 ppt) followed by ditches (16.3 ppt) and then wooded pools (5.8 ppt).

### 3.2. Adult Mosquito Sampling

A total of 399 adult females were collected representing four mosquito species (Table 3). All four species were also collected as larval in the marsh and wooded habitats (Tables 1 and 2). *Aedes sollicitans* was the most prevalent adult mosquito collected consisting of 71.7% (286/399) of all adults, followed by *Ae. taeniorhynchus* (20.1%, n = 80) and *Cx. salinarius* (14.8%, n = 59) (Table 3). Landing rates varied between sites with the EXP1-CON1-CON2 area having the highest landing rate at 15.1 females per minute.

### 3.3. Discussion

This study showed clear differences in the larval mosquito communities between different tidal salt marsh sites but no consistent patterns between sites with plugged ditches and control sites. Instead one plugged site (EXP1) and one control site (CON1), which were immediately adjacent to each other, had higher abundances than all other salt marsh sites, which had very few mosquitoes. As expected, *Ae. sollicitans* was the most common larval mosquito collected. *Aedes sollicitans* is considered the most common species in Atlantic coastal salt marshes [26]. *Aedes sollicitans* usually oviposits prior to flooding events of the marsh surface [26], which is consistent with our findings of it mainly in higher densities in panne habitats after spring tides. Plugged ditches in EXP1 were dominated by *Cx. salinarius. Culex salinarius* is often the most common *Culex* mosquito in salt marshes and has been collected from costal salt marshes at salinities ranging from 4.3 to 18.8 parts per thousand in previous studies (e.g., [27,28]). We collected *Cx. salinarius* in habitats with similar salinities, ranging from 4.4 to 13.7 ppt. *Culex salinarius* is commonly collected in heavily vegetated habitats [27]. By increasing tidal flushing, ditches can decrease anoxic stress and increase plant productivity near ditch banks [29]. In this study, ditches in EXP1 often had considerable vegetation along their edges (*pers. observation*). *Culex salinarius* can be common in impoundments where saltmarsh habitat has been reclaimed through dyking and flooding from upland runoff [19,30]. Ditch-plugging may create favorable environmental conditions for *Cx. salinarius* by altering abiotic factors, such as shade and salinity, and biotic factors, such as food resources and predators, and thus increase production of *Cx. salinarius*. Increasing abundances of *Cx. salinarius* could have important human health implications since *Cx. salinarius* can form large swarms of aggressive biters of both birds and humans, and thus can act as bridge vectors for encephalitis viruses in the surrounding area.

Wagner *et al.* [20] surveyed a variety of habitats, including saltwater bay marsh and flooded woodlands, at three wetland sites less than four miles from the sites in this study. Interestingly, *Cx. salinarius* and other species collected in this study, such as *Ae. taeniorhynchus*, *Ae. canator,* and *Anopheles bradleyi*, were not collected by them [20]. Wagner *et al.* [20] collected *An. quadrimaculatus* in saltwater bay marsh and *Ae. atlanticus*, *Ae. vexans*, and *Cx. territans*, which we did not collect in our study. Although only 15.1% of larval habitats were occupied by mosquitoes in our study, numbers of mosquitoes were high compared to those found by Wagner *et al.* [20]. Our study design randomly chose sampling locations so that we could rigorously compare sites rather than targeted sampling which would likely collect more mosquitoes but be subject to potential bias. Our data showed relatively higher densities in wooded areas, vegetated ditches, and pannes compared to ponds, and may indicate habitats where future studies should focus their sampling efforts to accurately characterize mosquito communities in coastal areas.

Total numbers of adults caught by landing counts were high at two of three wooded areas compared to those in other studies in Maryland that have used landing rate counts (e.g., [20]). The average landing rate count of 15.1 at the EXP1-CON1-CON2 wooded area is above the 12.0 average landing rate count threshold used by the Mosquito Control Section of the Maryland Department of Agriculture to determine when to apply insecticide to adults or larval habitats [31]. Salt marsh adult mosquitoes usually fly within 2–5 miles in search of bloodmeals, thus adults in our study are likely from a variety of coastal larval habitats [26]. The majority of mosquitoes in our study were collected from temporary pools in wooded habitats. Although these microhabitats had higher densities of mosquitoes compared to marsh habitats they represent a smaller wetland area that saltmarsh habitat, and thus are likely to have lower total mosquito production. We found that a higher proportion of pannes were occupied by *Ae. sollicitans* 4–5 days after spring tides. This result is consistent with past studies that have shown strong pulses of saltmarsh mosquito production following tidal events that often need control (e.g., [32,33]). Recently flooded habitats may be candidates for spray if targeted sampling showed that salt marshes within close proximity to human dwellings had mosquito densities consistently above this threshold. However, any application of spray would may pose risks to the salt marsh ecology and could be prohibitive in light of conservation and restoration goals.

Complex abiotic and biotic ecological processes, including microhabitat availability, inter- and intra-specific competition, and predation by macroinvertebrates and fish, could play critical roles in driving mosquito densities in salt marshes, and these processes may themselves be affected by ditch-plugging. Recent collections at the same sites as in this study indicate the common predatory fish *Fundulus heteroclitus* (mummichogs) and *Cyprinodon variegates* (sheepshead minnow) are common (Roman Jesien, Maryland Coastal Bays Program, *unpublished data*). The low numbers of larvae we found at CON2, CON3, and EXP2 in our study may be because fish at these sites have access to major mosquito habitats. Past authors have suggested that inter-site differences in marsh soils, salinities, elevation and species assemblages are important considerations when implementing mosquito control in salt marshes [29]. Little research has investigated these processes in salt marshes and how they may be affected by ditch-plugging but it is needed to better understand the management of key vector species and the diseases they carry.

Salt marshes provide numerous ecosystem services vital for human health and well-being, such as storm and shoreline protection, nutrient removal, fish and shrimp nurseries, food production, fur trapping and recreation (e.g., birdwatching, hunting) [1], which have been valued at close to $15,000 per ha per year worldwide [34]. Their role as abundant larval habitat for pest and vector mosquitoes warrants careful consideration of how restoration activities may alter disease risk. Past studies have repeatedly isolated EEEv and WNv from *Ae. sollicitans* [12,35] and *Cx salinarius* [36,37], and occasionally from *Ae. taeniorhynchus*, *Ae. cantator*, and the *An. crucians* complex of which *A. bradleyi* is a member [12,15,38]. Eastern Equine Encephalitis (EEEv) circulates enzootically in a wide variety of wild birds by other mosquitoes (e.g., *Culiseta melanura* and *Cs. morsitans*), but human transmission of EEEv in coastal areas in the northeastern U.S.A. usually occurs by *Ae. sollicitans* and *Cx. salinarius* which aggressively bite both birds and mammals [19,26]. Although human cases of EEEv are infrequent [16], mortality from encephalitic infections have been estimated to be 50–75%, which makes EEE the most severe insect-borne disease in North America. More mild systemic EEEv infection is characterized by chills, fever, malaise, arthralgia, and myalgia and lasts 1 to 2 weeks [39]. Symptoms in encephalitic patients are fever, headache, irritability, restlessness, drowsiness, anorexia, vomiting, diarrhea, cyanosis, convulsions, and coma [39]. Death usually occurs 2 to 10 days after onset of encephalitic symptoms but can occur much later [39]. Of those patients who recover, many are left with disabling and progressive mental and physical sequelae, which can range from minimal brain dysfunction to severe intellectual impairment, personality disorders, seizures, paralysis, and cranial nerve dysfunction [39]. West Nile virus (WNv) is also principally a disease of birds, but has infected over 30,000 people and caused over 1,200 deaths in the U.S. to represent the most medically important mosquito-borne disease in North America [18]. Most persons who become infected with WNv develop no clinical illness or symptoms [40]. Of the approximately 20% of infected people who do develop symptoms, most develop what has been termed West Nile fever, which is most commonly characterized by fever, headache and fatigue [40]. When WNv affects the central nervous system, it leads to encephalitis, headache, high fever, neck stiffness, stupor, disorientation, coma, tremors, convulsions, muscle weakness, and paralysis [40].

Eastern Equine Encephalitis (EEEv) and WNv infections incur substantial costs on public health systems and significant socioeconomic burden on broader society. The average cost per case of WNV illness has been estimated to be $US 34,200 and costs of lifelong disability have been estimated to be over >$3 million for both WNv and EEEv [41,42]. Outbreaks of WNv have been estimated to cost $20.1–42.3 M [42,43], and include non-medical costs related to days of work and lost productivity of $9.2 M. Because mild infections of WNv yield influenza-like symptoms, lost productivity and days of work due to saltmarsh mosquitoes are likely severely underestimated. The greatest public health and socioeconomic burden of coastal mosquitoes is probably represented by the considerable ongoing efforts to control the nuisance biting. Almost all coastal mosquito control programs in the U.S. were originally established to control the nuisance biting of *Ae. sollicitans*, *Cx. salinarius*, and *Ae. taeniorhynchus* [26].

Worldwide, salt marshes provide habitat for numerous other disease-vectoring mosquitoes, thus changes in the abundance and distribution of mosquitoes in salt marshes have global public health importance. For example, in Australia, Ross River virus (family Togoviridae, genus *Alphavirus,* RRv) infects an average of 500 people per year and coincides with production of the common tidal saltmarsh mosquitoes, *Aedes camptorhynchus*, and *Ae. vigilax* [44]. Because salt marshes are accessible abundant habitat worldwide and often in close proximity to seaports they can offer important habitat to help exotic mosquito species and pathogens establish and spread in new ranges. *Ochlerotatus camptorhyncus,* was first detected in New Zealand salt marshes in 1998, and posed a substantial threat of facilitating the invasion of Ross River Virus into New Zealand before its eventual eradication in 2010. The introduction and rapid dispersal of WNv since 1999 has demonstrated the infectious threat of mosquito-borne diseases. .*Aedes sollicitans, Cx. salinarius,* and *Ae. taeniorhynchus* are all efficient vectors of Rift Valley Fever virus (family Bunyaviridae, genus *Phlebovirus*), with *Ae. taenoirhynchus* among the species with the highest vector potential [45]. This pathogen is transmitted by *Aedes* mosquitoes in sub-Saharan Africa, but in 2000 RVF was first recorded outside of Africa, in Saudi Arabia and Yemen, and is a threat to establish and spread in the eastern U.S.A. [45].

## 4. Conclusions

This is one of the few studies that have quantified and compared mosquito species between salt marshes and neighboring habitats and between salt marshes with plugged ditches and salt marsh control sites on the Delmarva Peninsula. Our findings suggest that ditch plugging may alter mosquito species composition and overall productivity and that neighboring wooded sites may provide considerable habitat for salt marsh disease vectors. Future research will test the effects of ditch plugging on the ecology of salt marshes across multiple treatment salt marshes, control salt marshes, and upland sites, before and after treatments, to determine if alterations have a consistent impact on regional mosquito production. In light of the recent emergence of WNv and EEv in the northeastern U.S.A., and the importance of salt marshes in providing ecosystem services and habitat for vector mosquitoes worldwide, this research will help inform coastal restoration efforts to improve human health.

## Figures and Tables

**Table 1.** Larval abundances by mosquito species, proportion of mosquito-positive sampling occasions, and total densities from different habitats (numbers sampled in parentheses) at two plugged and three control tidal salt marshes from July to September, 2009. M, marsh pannes; D, ditches; P, ponds. EXP1 and CON1 were not independent hydrologic units. Numbers of sampling sites are shown in parentheses. Zero values are not recorded for clarity.

	**Control Site**									**Experimental (Plugged) Site**					
	**CON1**			**CON2**		**CON3**			**EXP1**			**EXP2**		
			
**Species**	**M (45)**	**D (9)**	**P (9)**	**M (45)**	**D (8)**	**M (80)**	**D (24)**	**P (9)**	**M (90)**	**D (27)**	**P (9)**	**M (80)**	**D (19)**	**P (24)**
*Aedes sollicitans*	159	4		2		1			28	66				
*Ae. taeniorhynchus*	6								1					
*Ae. cantator*	12	4							12	89				
*Culex salinarius*									8	229				
*Anopheles bradleyi*	31	43	24						87	128	7	1		
Total mosquitoes	208	51	24	2		1			136	512	7	1		
Proportion mosquito-positive occasions	0.36	0.56	0.33			0.02			0.21	0.26	0.33	0.01		
Density per dip (± SE)	1.670 (0.906)	0.887 (0.484)	0.133 (0.084)	0.089 (0.089)		0.003 (0.003)			0.468 (0.175)	7.70 (5.30)	0.036 (0.020)	0.003 (0.003)		

**Table 2.** Larval abundances by mosquito species, total proportion of mosquito-positive sampling occasions, and densities of total mosquitoes from habitats in three wooded areas bordering the upland edges of salt marshes from July to September, 2009. Numbers of sampling sites are shown in if parentheses. Zero values are not recorded for clarity.

**Species**	**EXP1-CON1-CON2 (37)**	**EXP2 (22)**	**CON3 (20)**
*Aedes sollicitans*	492	348	24
*Ae. taeniorhynchus*	162	152	
*Ae. cantator*	107	52	8
*Culex salinarius*	1	60	
*Anopheles bradleyi*	31	65	3
*Psorophora ferox*		10	
Total mosquitoes	793	687	35
Proportion mosquito-positive occasions	0.41	0.50	0.20
Density per dip (±SE)	6.62 (2.81)	7.31 (3.17)	0.44 (0.20)

**Table 3.** Numbers of adult mosquitoes collected by species using landing rate counts from 3 wooded areas that border the upland edges of coastal salt marshes. Landing counts were conducted over three nights in September 2009.

**Species**	**EXP1-CON1-CON2**	**EXP2**	**CON3**
*Ae. sollicitans*	132	101	23
*Ae. taeniorhynchus*	53	24	3
*Cx. salinarius*	25	34	
*An. bradleyi*	2	2	
Total mosquitoes	212	161	26
Total collection time	14	15	24
Total landing rate per minute	15.1	10.7	1.1
